# OFGPMA: Optimal frequency graph representation learning for pseudogene and miRNA association prediction

**DOI:** 10.3389/fgene.2025.1643921

**Published:** 2025-11-26

**Authors:** Yongbin Zeng, Lixiang Xiong, Yungui Luo

**Affiliations:** 1 College Information Science and Engineering, Wuchang Shouyi University, Wuhan, China; 2 College of Information Engineering, Wuhan Huaxia Institute of Technology, Wuhan, China

**Keywords:** optimal frequency graph, global random walk with restart, local enclosing subgraph, graph representation learning, pseudogene and miRNA association prediction

## Abstract

Pseudogenes are genomic segments that resemble functional genes structurally yet remain biologically inactive. MicroRNAs (miRNAs), a subclass of non-coding RNAs, are critical regulators of various cellular mechanisms. These pseudogenes and miRNAs interact mutually, forming competitive endogenous RNA (ceRNA) networks alongside mRNA to influence physiological processes. Such regulatory networks have been implicated in numerous pathological conditions. Consequently, investigating pseudogene-miRNA associations holds promise for advancing disease diagnostics. Nevertheless, existing approaches to identify these relationships predominantly rely on labor-intensive experimental techniques, demanding substantial time and financial investments. Consequently, developing an effective computational framework that can identify new pseudogene-miRNA associations (PMAs) is crucial. To this end, we propose an optimal frequency graph representation learning framework named OFGPMA, for pseudogene-miRNA association prediction. OFGPMA enhances graph neural network expressiveness by learning both high-frequency energy and low-frequency energy components within the pseudogene-miRNA bipartite graph, utilizing Rayleigh and Chebyshev pooling techniques. This approach captures the graph’s global topology via Random Walk with Restart (RWR) and identifies potential local substructure features through enclosing subgraph analysis, thereby achieving a more comprehensive integration of the entire graph information. Comprehensive experiments show that OFGPMA outperforms state-of-the-art methods in terms of performance, while also exhibiting excellent generalization capabilities.

## 1 Introduction

Pseudogenes, also known as false genes, are non-functional remnants formed during the evolution of gene families (Carninci et al., 2005; Shi et al., 2016). They are similar to normal genes but are DNA sequences that have lost their normal functions and are often found in multi-gene families of eukaryotes (Setoyama et al., 2011; Ma et al., 2021). MiRNA is one type of non-coding RNA, with lengths between 19 and 25 nucleotides, and they account for roughly 3% of the genome (Hydbring and Badalian-Very, 2013; Liu et al., 2016). Predicting the correlation between the two is of crucial significance for revealing gene regulatory networks, disease mechanisms and the development of precision medicine (Zhang et al., 2012; Stiegelbauer et al., 2014). A large number of studies have demonstrated that pseudogenes and miRNAs interact with each other and, together with mRNA, form a ceRNA network. This network plays a role in regulating biological processes and is associated with various diseases. Predicting pseudogene-miRNA associations can provide advisory treatment plans for some difficult and complicated diseases (Salmena et al., 2011; Rutnam et al., 2014; Karreth et al., 2015).

Present miRNA-related databases only offer fundamental information about miRNAs, such as their target genes and genomic locations. Details regarding their connections to diseases, which are crucial for understanding disease mechanisms, are often overlooked. Thankfully, some researchers have begun to recognize the significance of pseudogene–miRNA associations (PMAs) and have compiled the currently known associations into databases. For example, starBase v2.0 (Li et al., 2014) includes 444 pseudogenes and 173 miRNAs, which permits the exploration of their interactions through computational approaches. However, most discoveries of PMA are dependent on biological experiments that are not only time-intensive and resource-demanding but also constrained by the limited number of confirmed PMAs. On the other hand, predicting novel associations between pseudogenes and miRNAs via computational methods facilitates screening of potential PMAs.

Graph signal processing (GSP) adapts signal processing concepts to graphs, encompassing operations such as sampling, convolution, and filtering in the spectral domain. Graph signals are defined as numerical or vector values on graph nodes (Ortega et al., 2018; Hu et al., 2022). To analyze these signals, GSP employs spectral decomposition of either the graph Laplacian or adjacency matrix, revealing their spectral characteristics. These characteristics describe how signal energy is distributed among various frequency components inherent to the graph’s topology (Dong et al., 2020). The spectral characteristics essentially describe the degree of fit between the graph signal and the graph topology: low-frequency energy information corresponds to a globally smooth signal distribution (similar values at adjacent nodes), while high-frequency energy information corresponds to local abrupt fluctuations in the signal (differences in values at adjacent nodes) (Sandryhaila and Moura, 2013; Gavili and Zhang, 2015; Ramakrishna et al., 2020). Recently, the concept of graph signal processing has found extensive use in the field of biological networks, mainly focusing on using graph structures to model and conduct in depth analysis of complex biological systems. For example, Peng et al. modeled the drug response of cancer cells as a hypergraph, and simultaneously applied low-frequency component and high-frequency components filters to the hypergraph, effectively extracting both common and differential features among the hypergraph nodes (Peng et al., 2025).

Current computational approaches leveraging similarity networks in biological applications commonly adopt a key assumption: given a known interaction between pseudogene and miRNA, functionally or structurally similar pseudogenes may also engage with correspondingly similar miRNAs. For example. Zhou et al. integrated pseudogene expression data and miRNA sequence features to construct three similarity networks, namely Jaccard, Cosine, and Pearson, and used Graph Autoencoder (GAE) to aggregate node features and network topological relationships to generate low-dimensional embedding representations (Zhou et al., 2021). Despite its available predictive performance, PMGAE merely utilizes the structural information of the graph itself and does not treat label information as supervisory signals, resulting in its single train pattern as a non-end-to-end mode. More importantly, the GAE within PMGAE is often limited by the vanilla GCN with two layers, making it challenging for it to aggregate the node features and topological. Moreover, PMGAE adopts pseudogene and miRNA similarity networks, but the similarity assumption maybe does not hold in the association network of pseudogenes and miRNAs. A widespread biological consensus is that minor nucleotide differences can lead to significant variations in the functions of the proteins transcribed and translated from them, which often casts doubt on the availability of the similarity assumption in biological networks.

Recently, owing to its superior performance in graph representation learning, subgraph-based GRL (SGRL) has become a representative method for link prediction (Frasca et al., 2025; Wu et al., 2025; Zeng et al., 2025; Bouritsas et al., 2020). Unlike prediction models based on the similarity assumption (such as PMGAE), SGRL only extracts closed subgraphs in bipartite graphs and overcame the limitations of similarity assumption (Zhang and Chen, 2019; Teru et al., 2020). For instance, Zhang et al. proposed a link prediction model SEAL on the basis of graph neural networks (GNN), which automatically learns heuristic features from local closed subgraphs to address the limitations of traditional predefined heuristic methods (Zhang and Chen, 2025). Motivated by this method, Xu et al. put forward a subgraph-based model and applied it to enhance the prediction of associations between enhancers and diseases, further improving the accuracy of candidate disease-related enhancers by capturing local closed subgraphs of enhancers and diseases (Xu et al., 2024). Wang et al. introduced an innovative method called KnowDDI for predicting drug-drug interactions (DDI). It can adaptively extract and optimize subgraphs related to specific drug pairs, thereby enhancing prediction accuracy and interpretability (Wang J. et al., 2023). Wang et al. proposed a meta-learning-based zero-shot drug-target interaction (DTI) prediction framework for proteins, with its core innovation being the introduction of a weakly supervised subgraph information bottleneck module. This method relies solely on global DTI labels and does not require pocket annotations. It can identify key subgraphs in protein structures as potential binding pockets by dynamically learning the node allocation matrix (Wang Y. et al., 2023). Swarnkar et al. proposed a method that integrates gene expression data with protein-protein interaction networks (PPI) to identify key disease-related gene modules by recognizing dense subgraphs (Swarnkar et al., 2015). These methods have all demonstrated the effectiveness of local subgraphs and the non-essentiality of the similarity assumption.

The above-mentioned methods overcome the limitations of similarity-based networks. Their inductive approach uses closed subgraphs to adaptively learn the local neighborhood subgraph information of the target node. However, from the perspective of extracting information from graph structure, the main limitation of their method lies in its insufficient capture of global topological features. Although relevant theories have demonstrated that local subgraphs can approximate high-order heuristics, its core mechanism still relies on the preset h-hop closed subgraph, which is essentially a compromise of a local perspective.

To this end, we introduce a novel optimal frequency graph representation learning for pseudogenes and miRNA interactions prediction (OFGPMA) to address the above problems. Our model consists of two modules: the optimal frequency discovery (OFD) module and the graph representation learning (GRL) module. To enhances the expressive power of graph neural networks, The OFD learn the optimal frequency energy features of graphs through aligning the high-frequency components and low-frequency components information of the graphs. Specifically, OFD explicitly enhances the high-frequency components information in the bipartite graph of pseudogenes and miRNAs through Rayleigh pooling, thereby accurately capturing the key features of the graph nodes. Meanwhile, it implicitly extracts the low-frequency components information of the graph through Chebyshev pooling, generating important representations that reflect the commonalities of each node. Ultimately, by fusing the high-frequency and low-frequency energy information, it simultaneously learns the difference and commonality information of the graph, while resulting in a fused graph with optimal frequency structure. Then, the GRL uses the fused graph for graph representation learning. We use the graph extracted by the random walk with restart (RWR) as the explicit topological structure and the topology subgraph obtained through enclosed subgraph representation learning as the corresponding latent substructure, with the goal of accommodating explicit global topology. In detail, we use the RWR algorithm to globally extract the full graph representation of pseudogenes as explicit topological features, and simultaneously extract the enclosed subgraph features of miRNAs as implicit substructure features. We then fuse the global features of pseudogenes with the local features of miRNAs. Through this method, we can not only overcome the limitations of single local features, but also effectively combine and balance global and local features. In summary, the key contributions of OFGPMA can be outlined as follows:

• The OFD focuses on the processing and optimization of graph signals in the frequency domain. By employing an original high-frequency/low-frequency separation, enhancement, and fusion strategy, it generates an optimal frequency graph structure, which significantly enhances the capability of node feature representation.
• The GRL focuses on comprehensively utilizing graph topological information by integrating topological features at two distinct scales: global (RWR) and local (enclosing subgraph). This approach overcomes the limitations of a single perspective, thereby achieving more comprehensive network structure modeling.
• The superior performance of OFGPMA is validated through comprehensive experiments. The importance of every component within the model is substantiated by ablation tests. Furthermore, case studies reveal OFGPMA’s capability to detect previously unknown pseudogene-miRNA interactions.

## 2 Materials and methods

### 2.1 Data collection

Currently, the only database that records the association between pseudogenes and miRNAs is starBase v2.0 (Li et al., 2014). We get the association data of pseudogene-miRNA pairs from the starBase database and preprocessed it using the same data processing method as Zhou et al. Ultimately, we obtained the data including 444 pseudogenes, 173 miRNAs and 1,884 pseudogene-miRNA pairs.

### 2.2 Overview of OFGPMA

**FIGURE 1:**
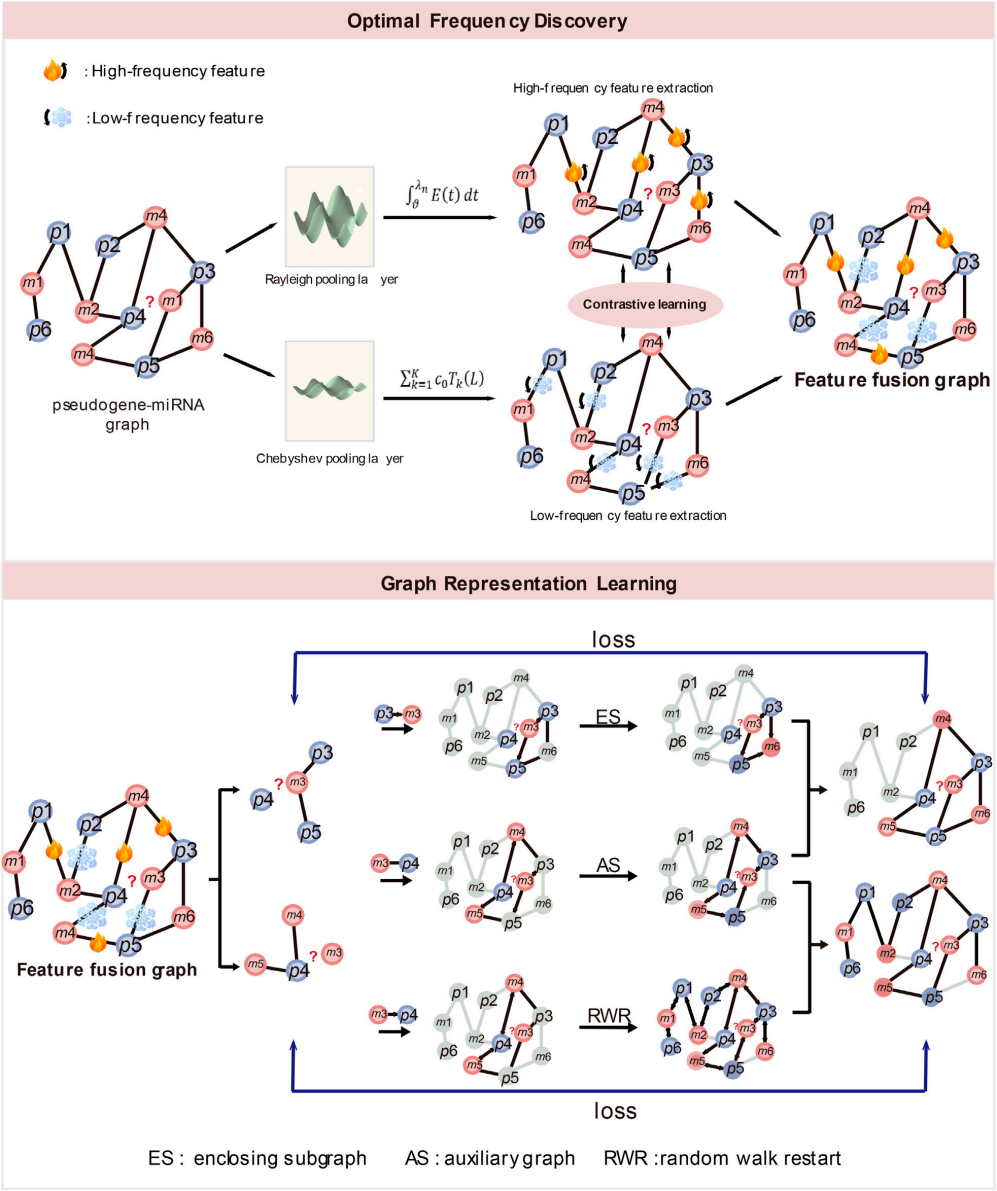
(1) The structure of optimal frequency discovery (top). (2) The structure of graph representation learning (bottom). ES denotes enclosing subgraph, AS denotes auxiliary graph and RWR denotes random walk restart.

Firstly, we set pseudogene-miRNA association pairs as a bipartite graph $G=\left\{V,\xi \right\}$ , V is node set and ξ is edge set. Specifically, V includes pseudogene node $P=\left\{p_{1},p_{2},\ldots ,p_{N}\right\}$ and miRNA node $M=\left\{m_{1},m_{2},\ldots ,m_{A}\right\}$ . ξ includes pseudogene-miRNA association pairs. Then, we introduce an optimal frequency graph representation learning framework named OFGPMA to infer novel PMAs (Figure 1). Our model mainly comprises of two parts: 1) optimal frequency discovery, which includes Rayleigh pooling and Chebyshev Pooling around the pair ( $p_{i}$ , $m_{i}$ ); 2) graph representation learning, which employs graph-level GNN to learning the embeddings of local enclosing subgraph and global RWR graph.

The main notations used in this paper are summarized in Table 1.

**TABLE 1:** Main notations used in this study.

Notation	Description
miRNAs	microRNAs
ceRNA	Competitive endogenous RNA
PMA	Pseudogene-miRNA association
GSP	Graph signal processing
GNN	Graph neural network
Graph Representation Learning	GRL
SGRL	Subgraph-based GRL
OFD	Optimal frequency discovery
RWR	Random walk with restart

### 2.3 Node representation

MiRNA sequence data is represented as a string composed of four nucleotides. In this paper, we use k-mer to represent miRNA sequences as a 64-dimensional feature vector, where k=3 . Similarly, pseudogenes are processed in the same way. The final feature matrix dimension of pseudogenes (P) is 444 × 64, and that of miRAN (M) is 173×64 . For specific details, please refer to Supplementary Materia Section 1.

### 2.4 Optimal frequency graph discovery

In the OFG module, the Rayleigh pooling is proposed to extract the high-frequency energy information of the pseudogene and miRNA bipartite graph, and use the Chebyshev wavelet transform to learn the low-frequency energy information of the bipartite graph. Subsequently, by integrating the high-frequency and low-frequency energy features while jointly capturing the distinct and shared patterns within the graph, we derive a fused graph that exhibits the most favorable frequency configuration. Through this approach, OFG can significantly enhance the GNN’s ability to express graph structure information.

#### 2.4.1 Rayleigh pooling

The Rayleigh Quotient is an important concept in signal processing, used to characterize the energy distribution of graph signals on the Laplacian matrix (Li, 2004). Specifically, the Rayleigh Quotient reflects the weighted cumulative energy of graph signals at all frequencies. To explicitly extract the high-frequency component spectral information of the graph, we improved the method in (Dong et al., 2023) by introducing two parameters ϑ and μ , thereby enabling Rayleigh pooling to be more inclined to capture high-frequency energy information and assign higher weights to high-frequency features. In this way, not only can the contribution of high-frequency components be amplified, but also features containing high-frequency energy information can be effectively distinguished. Through this method, the high-frequency features $H_{RQ}$ of the entire graph can be extracted. For specific details, please refer to Supplementary Materia Section 2.

The Rayleigh Pooling helps to identify significant changes within the graph structure and improves the model’s capacity for PMA prediction by emphasizing the signal components with the greatest information content.

#### 2.4.2 Chebyshev pooling

Meanwhile, the Chebyshev Wavelet Transform (CWT) is designed to extract the low-frequency features of graphs. The Chebyshev Wavelet Transform is an efficient multi-scale graph signal analysis tool. Its main objective is to capture the multi-band energy characteristics in the graph structure while avoiding the computational bottlenecks existing in traditional spectral methods (Du et al., 2017). As a spectral domain filtering method based on polynomial approximation, the core idea of the Chebyshev Wavelet Transform lies in designing multiple wavelet filters to cover different frequency ranges. The Chebyshev wavelet transform realizes a learnable low-pass component filter through polynomial approximation. The specific implementation details are provided in Supplementary Materia Section 3. Through this method, we can ultimately obtain the low-frequency features $H_{CWT}$ of the entire graph.

#### 2.4.3 Information fusion

After undergoing Rayleigh pooling and Chebyshev pooling, we can obtain the high-frequency energy information $H_{RQ}$ and low-frequency energy information $H_{CWT}$ of the pseudogene and miRNA network. Then, we use information fusion strategy calculated as the embeddings *Embedding:*

$$Embeddings=\pi H_{RQ}+\left(1-\pi \right)H_{CWT}$$ (1)

where π is the scaling factor and indicates that the model focuses on energy information of different frequencies.

### 2.5 Graph representation learning

For the graph representation learning of pseudogenes-miRNA pair, there are three steps: 1) miRNAs subgraph extraction. For miRNAs, a closed subgraph representation learning based on local structural features is adopted. 2) RWR graph extraction. For pseudogenes, a random walk restart (RWR) method based on global structural attributes is used. 3) encoder layer. GNN is employed to generate the embeddings of the extracted graph representations, and information fusion is conducted to obtain concise edge embeddings.

#### 2.5.1 miRNA subgraph extraction

For miRNA, we adopt a closed subgraph representation learning based on local structural features. The extraction of the closed subgraph of miRNA can be divided into two steps: First, construct the main graph $G_{\left(m,p\right)}^{k}$ with the miRNA nodes as the starting points; second, based on the pseudogene auxiliary nodes related to the pseudogenes, extract the auxiliary subgraph $G_{\left(a,p\right)}^{k}$ . Finally, the two subgraphs are merged to jointly form a local closed subgraph for miRNA $G_{m}^{k}=G_{\left(m,p\right)}^{k}\cup G_{\left(a,p\right)}^{k}$ . Starting from the miRNAs, we iteratively expand the pseudogene nodes within 1-hop and 3-hop to form the closed subgraphs $G_{\left(m,p\right)}^{k}$ of the miRNAs. For instance, for the path (*m*→*p*1→*m*1→*p*) and (*m*→*p*1→ *m*1→*p*2→*m*2→*p*3→*m*), starting from miRNA nodes, extract the adjacent pseudogene nodes to construct a local closed subgraph. It can be observed that in both of these two paths, the odd-numbered jump neighbor nodes of miRNA are all pseudogenes. Algorithm 1 builds a local subgraph by iteratively expanding the k-hop neighbors of pseudogene node preserving the topological structure closely related to the target while avoiding the interference of the target edge on the prediction. This process provides the subsequent graph neural network with rich semantic local context information.

Finally, Algorithm 1 integrates motif path information to enhance local topological coverage. After iterating Algorithm 1 for R times, the subgraph range is gradually expanded to ensure full coverage of the high-order neighbors of the target node.

$$R=\lfloor \frac{v\sqrt{e}}{2}\rfloor$$ (2)

where v and e represent the count of nodes and the count of edges in a bipartite graph *G*, respectively.

Algorithm 1 Enclosing Subgraph extraction.

1: **Input:** bipartite graph *G*, pseudogene-miRNA pair (m, p), the count of *k*
2: **Output:** enclosing subgraph $G_{\left(m,p\right)}^{k}$ or auxiliary subgraph $G_{\left(a,p\right)}^{k}$ about pseudogene-miRNA pair (m, p)
3: $M=\left\{m\right\}P=\left\{p\right\}$

4: **for** i=1,2,…,k **do**
5: Find all new miRNA nodes set $M_{new}$ directly connected to the current pseudogene set P, excluding existing nodes.
6: Find all new pseudogene nodes set $P_{new}$ directly connected to the current miRNA set M, excluding existing nodes.
7: P = P $\cup P_{new}$

8: M = M $\cup M_{new}$

9: Construct subgraph $G_{\left(m,p\right)}^{k}$ or $G_{\left(a,p\right)}^{k}$ by utilizing node sets P , M

10: **end**
11: Remove edge (m, p) need to be predicted from $G_{\left(m,p\right)}^{k}$ or $G_{\left(a,p\right)}^{k}$

#### 2.5.2 RWR graph extraction

To analyze pseudogenes, we employ a random walk with restart (RWR) approach that utilizes global network topology. The algorithm initiates traversal from pseudogene nodes, systematically identifying miRNA nodes located at odd-hop distances. This process progressively extends to encompass all miRNA nodes within the complete network, enabling comprehensive characterization of pseudogene relationships across the entire graph:

$$\rho =cAD^{-1}\rho +\left(1-c\right)e$$ (3)

where $c\in \left(0,1\right)$ is restart probability, ρ is adaptive parameters with $\rho_{i}$ denoting the probability at node *i.* For miRNA nodes, since RWR samples the pseudogene-associated miRNA nodes, h-hops is an odd number, ensuring that each sampled node is a miRNA. The restart probability represents that the probability of choosing a neighbor for the next hop is c, and the probability of returning to the starting point is (1-c). e denotes starting vector and if *i* is starting node, $e_{i}$ is set 1 else set 0. Thus, the starting vector *e* allows us to preserve the node’s local topological structure and $AD^{-1}$ allows us to further visit their neighborhoods. After RWR graph extraction, we can obtain a global graph $G_{RWR}$ from pseudogene sampling.

### 2.6 Encoder layer

To cover the neighborhood information of both the local encolsing subgraph and the RWR global graph, we merge the two graphs $G_{m}^{k}$ and $G_{RWR}$ . Next, we use two layers of GCN to learn topological features for $G_{m}^{k}$ and $G_{RWR}$ . Finally, we can get embeddings $Z^{new}$ . For specific details, please refer to Supplementary Materia Section 4.

### 2.7 Model optimization

The contrastive learning loss function is used to calculate the gap between $H_{RQ}$ and $H_{CWT}$ :

$$L_{cl}=-\frac{1}{2\left|V\right|}\left(\sum_{v\in V}\log\Gamma \left(H_{RQ},H_{CWT}\right)+\sum_{v\in V}\log\left(1-\Gamma \left(H_{RQ},H_{CWT}\right)\right)\right)$$ (4)

where Γ () is the contrastive discriminator constructed by a simple bilinear function that estimates similarities between $H_{RQ}$ and $H_{CWT}$ . We use Kullback-Leibler (KL) divergence to calculate loss between $Z^{p}$ and $Z^{RWR}$ :

$$L_{kl}=KL\left(Z^{m},Z^{RWR}\right)=\sum \log_{2}\frac{\;Z^{m}}{Z^{RWR}}$$ (5)

The binary cross-entropy loss is employed to optimize OFGPMA:

$$L_{bce}=-\frac{1}{N}\sum Ylog\hat{Y}+\left(1-Y\right)\log\left(1-\hat{Y}\right)$$ (6)

where *N* is the number of all pseudogene-miRNA pairs in the batch. Y and $\hat{Y}$ are the ground truth and prediction score, respectively. Coupled with the $L_{cl}$ and $L_{kl}$ , OFGPMA can be trained by minimizing the final loss which can be calculated as:

$$Loss=\left(1-a-\beta \right)L_{cl}+L_{kl}+L_{bce}$$ (7)

where α and β are learnable parameters. The pseudo-code of OFGPMA as follows:

Algorithm 2 OFGPMA train description.

1: **Input:** training set pseudogene-miRNA pairs, k-hops;
2: **Output:** the convergent training model OFGPMA;
3: Randomly initialize model parameters;
4: Construct a bipartite graph G;
5: **Repeat**
6: Generate a fused graph Gf by Equation 1 and supplementary materials Equations 1–9 from G;
7: Samples miRNA enclosing subgraph and pseudogene RWR graph from $G_{f}$ ;
8: Upgrade miRNA and pseudogene representations with two-layer GCN;
9: Update model parameters by minimizing the loss in Equation 7;
10: Training process terminates when the model converges or all epochs are completed;
11: **Return** the train OFGPMA;

## 3 Results

### 3.1 Evaluation criteria

In OFGPMA. we employ frequently five evaluation metrics to evaluate its performance, including AUC, AUPR, PREC, REC and F1-score. AUC denotes the area under the Receiver Operating Characteristic (ROC) curve, AUPR indicates the area under the Precision-Recall (PR) curve, PREC refers to precision, and REC stands for recall., respectively. For the specific calculation formula, please refer to Supplementary Materia Section 5.

### 3.2 Performance of OFGPMA

To assess the performance of OFGPMA, we conducted five-fold cross-validation (5-CV). Specifically, experimentally validated pseudogene-miRNA interactions were used as positive samples. An equal number of negative instances were randomly selected from unconfirmed pseudogene-miRNA pairs. The final dataset for the 5-CV experiments was formed by combining these positive and negative samples.

The 5-CV methodology entailed the random division of the data into five distinct subsets. During each iteration, a single subset was designated as the test set, with the other four subsets combined to form the training set. Importantly, random partitioning ensured that both training and test data within each fold maintained an equal balance of positive and negative samples. To account for variability and minimize bias in the 5-CV findings, performance metrics were averaged over all folds, and their standard deviation was calculated. It should be noted that although AUC summarized overall model efficacy, AUPR furnished a more nuanced perspective (Ling et al., 2025). Consequently, AUC and AUPR were utilized as the principal performance indicators.

As presented in Table 2, OFGPMA achieved an AUC score of 0.8718 and an AUPR score of 0.9105 across the five folds. Performance variations were observed: the third fold yielded a lower AUC value compared to other folds, while the first fold exhibited a higher AUPR value. These fluctuations were attributable to model performance variability induced by different random seeds. Throughout the cross-validation, Precision and Recall metrics demonstrated minor oscillations around their respective means, with an overall limited range of variation. Collectively, these robust results confirmed the potential utility of OFGPMA for predicting potential PMAs.

**TABLE 2:** Performance of OFGPMA.

Fold	AUC	AUPR	Precision	Recall
1	0.8711	0.9118	0.9230	0.8993
2	0.8635	0.8996	0.9193	0.9103
3	0.8613	0.9110	0.9217	0.9005
4	0.8767	0.8998	0.9189	0.8996
5	0.8678	0.9007	0.9218	0.9076
Mean	0.8718	0.9105	0.9211	0.9015

### 3.3 Comparison experiment

The efficiency of OFGPMA was assessed through two comparative approaches: 1) direct comparison with specialized PMA predictors such as PMAGAE; 2) Secondly, comparison with diverse computational models including random walk, deep learning, and matrix factorization frameworks, alongside models designed for other biomedical entity associations. Each model was evaluated via 5-fold cross-validation using our dataset, with final scores representing the mean values computed over 100 experimental iterations.

#### 3.3.1 Comparison with PMAGAE

**FIGURE 2:**
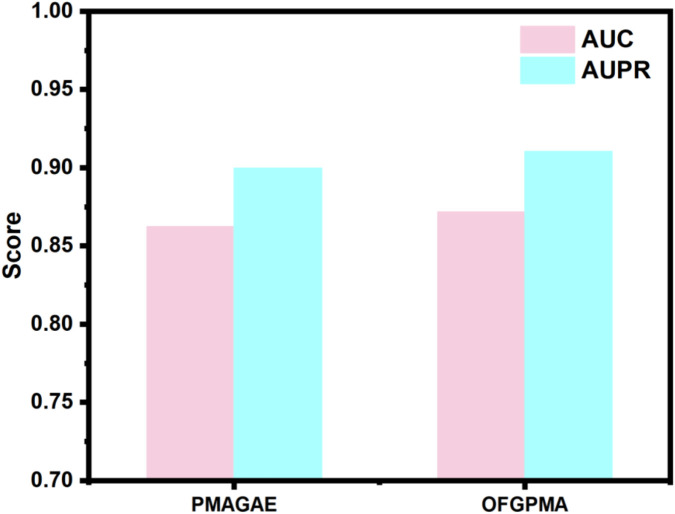
Model performance of PMAGAE and OFGPMA.

In the first comparison method, we compared OFGPMA with PMAGAE. PMAGAE is the first proposed computational model for predicting the association between pseudogenes and miRNAs. It is based on the similarity network of pseudogenes and miRNAs and is specifically designed for identifying PMAs. PMAGAE leverages the similarities between pseudogenes and miRNAs and calculates the association strength by integrating the similarity features and connections of nodes using GAE (Zhou et al., 2021). To ensure equitable comparison, we re-implemented PMAGAE under identical random seed conditions. Comparative results (Figure 2) reveal PMAGAE’s AUC (0.8623) and AUPR (0.8996), aligning with prior literature yet demonstrating inferior performance relative to OFGPMA.

#### 3.3.2 Comparison with other baselines

In our comparative study, we conducted performance evaluations between OFGPMA and nine existing graph neural network approaches that represent current methodological standards. The compared techniques are detailed in the following listing.

• Node2Vec (Grover and Leskovec, 2016): Node2Vec formulates node embedding as an optimization challenge, employing a neighborhood sampling strategy that harmonizes local and global network exploration via a tunable random walk process. The algorithm’s flexibility stems from its adjustable bias parameters governing walk behavior.
• GCN (Kipf and Welling, 2016): GCN, as a semi-supervised framework, generates node embeddings through direct processing of graph adjacency matrices. The model operates on the pseudogene-miRNA bipartite network in its raw topological form, deliberately excluding supplementary biological feature to maintain architectural purity.
• GAT (Veličković et al., 2017): GAT enhances graph processing through attention mechanisms, where node relationships are dynamically weighted. The pseudogene-miRNA bipartite graph serves as direct input to the attention-based predictor for uncovering previously unknown biological relationships.
• GIN (Xu et al., 2018): Renowned for its discriminative power in graph-based prediction, GIN processes the fundamental pseudogene-miRNA network structure to hypothesize new functional associations between these molecular entities. The architecture demonstrates particular efficacy in biological network inference tasks.
• NMFMC (Zheng et al., 2022): NMFMC employs non-negative matrix decomposition to reconstruct incomplete association matrices, enabling the discovery of previously uncharacterized pseudogene-miRNA interactions. The derived predictions serve as valuable comparative data for subsequent validation studies.
• ERMDA (Dai et al., 2022): Through an ensemble learning framework, ERMDA constructs multiple balanced training datasets while learning hierarchical feature representations. Originally designed for miRNA-disease prediction, the algorithm demonstrates transfer learning capability when applied to pseudogene-miRNA network analysis.
• NIMGSA (Jin et al., 2022): Combining graph autoencoder architecture with attention mechanisms, NIMGSA performs neural matrix imputation for biological relationship prediction. The framework demonstrates particular effectiveness when processing sparse pseudogene-miRNA interaction data.
• CGHCN (Liang et al., 2024): CGHCN integrates conventional graph convolution with hypergraph neural operations, capturing both pairwise and higher-order relationships within biological networks. The model excels at identifying complex interaction patterns in omics data.
• MSHGANMDA (Wang S. et al., 2023): Utilizing meta-subgraph representations within an attention-based graph neural framework, MSHGANMDA provides enhanced prediction of molecular interactions. Its architectural flexibility allows direct application to pseudogene-miRNA association mining tasks.

**FIGURE 3:**
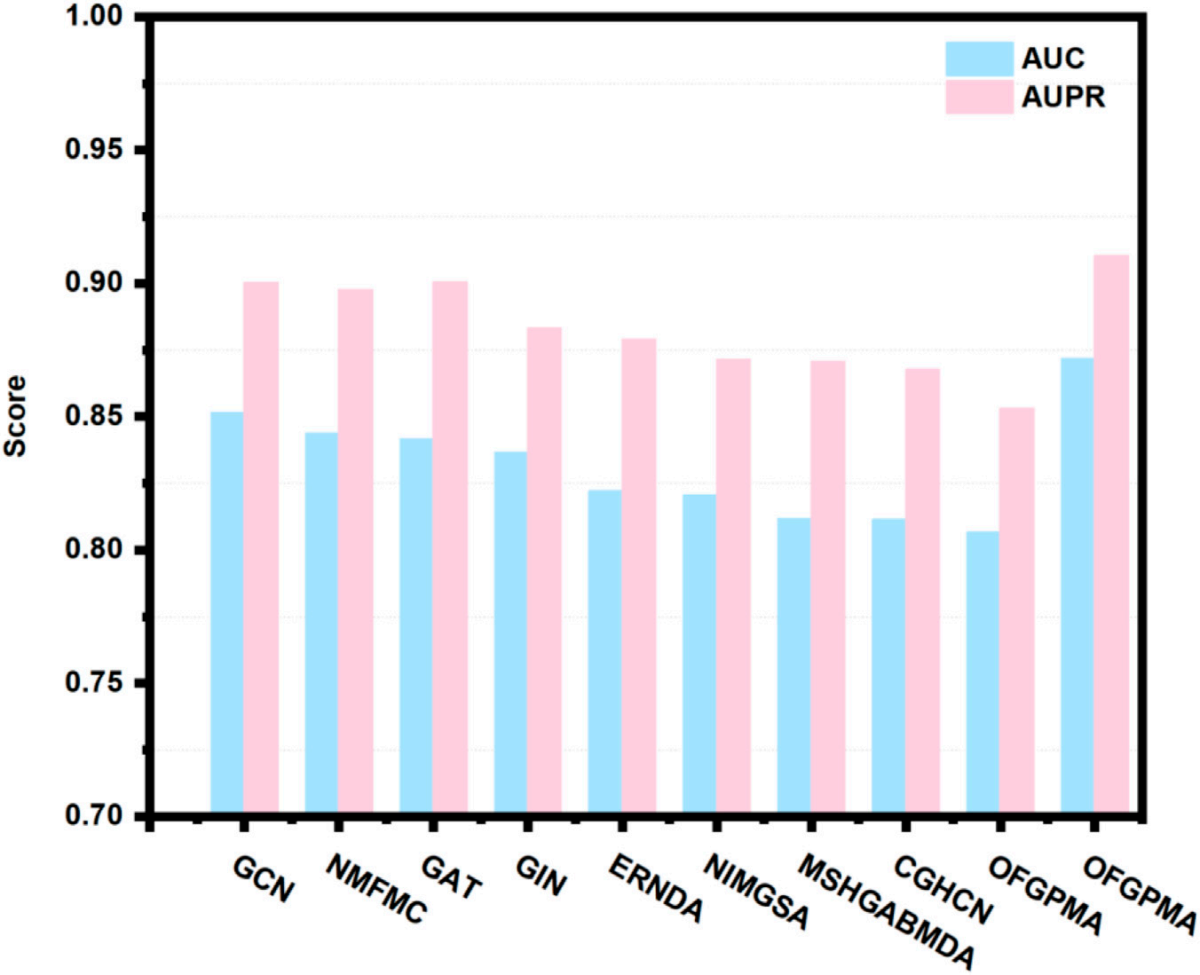
AUC and AUPR value of OFGPMA as well as the other nine baseline models.

Using 5-fold cross-validation and AUC/AUPR scores as primary metrics, we evaluated the proposed OFGPMA model against nine existing approaches. Figure 3 illustrates that OFGPMA achieved superior performance in both AUC and AUPR compared to all other models. On the starBase dataset, OFGPMA notably achieved an AUC value of 0.8718. GCN followed as the second-best performer, though a 2.03% performance gap separates it from OFGPMA, confirming our model’s significant contribution to improving graph neural network expressiveness. The third-ranked model, NMFMA, while reinforces that local structural information (captured by enclosing subgraphs) is valuable for PMA prediction, OFGPMA’s integration of global RWR graph context with local information yields demonstrably stronger results. Since the closed subgraph only captures the local subgraph information of the pseudogene and miRNA bipartite graph, the OFGPMA method, by integrating the global RWR graph information with the local closed subgraph information, can more comprehensively represent the information of the entire graph, which is of great significance in information integration. CHGCN performed the worst among the other nine models, indicating its lower applicability in the PMA prediction task. Collectively, OFGPMA achieves top performance across all evaluated metrics on the starBase dataset, confirming its strong competitive edge. This enhancement is credited to the elaborate Rayleigh pooling, Chebyshev pooling, and global RWR strategy, which can more comprehensively represent the information of the entire graph and capture efficient global topological semantics, respectively.

### 3.4 Robustness analysis

An optimal predictive model is expected to exhibit strong robustness and generalization capabilities. To assess the generalization potential of OFGPMA and confirm its broader applicability, this work applied it to several distinct association prediction tasks. Specifically, multiple datasets encompassing miRNA-disease, gene-disease, piRNA-disease, and microbe-disease associations were compiled. The specific data processing procedures are detailed in the Supplementary Materia Section 6. The specific data quantities are shown in Table 3.

**TABLE 3:** Datasets on miRNA-disease, gene-disease, piRNA-disease, and microbe-disease associations.

Pair	Type	Number
miRNA- disease (Li et al., 2021)	miRNA	156
	disease	187
	interaction	1,983
Gene-disease (Luo et al., 2019)	gene	2,909
	disease	1,154
	interaction	4,432
piRNA-disease (Chen et al., 2024)	piRNA	4,976
	disease	28
	interaction	7,939
Microbe-disease (Wang et al., 2023b)	microbe	1,177
	disease	134
	interaction	4,499

**FIGURE 4:**
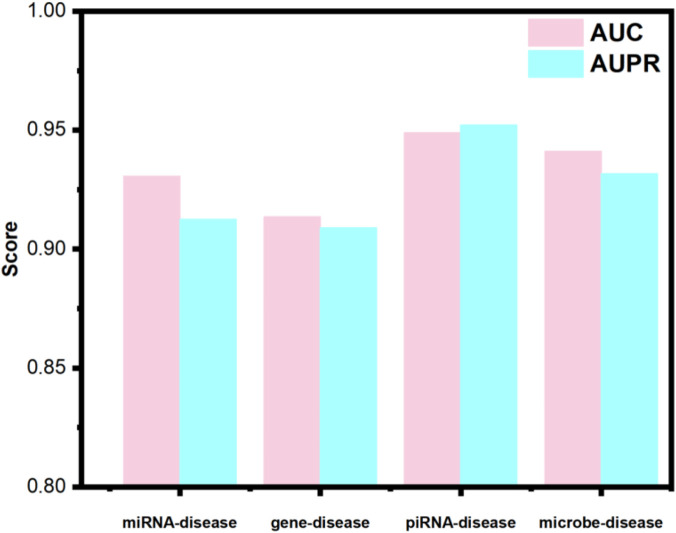
Performance of OFGPMA for predicting different data types.

Utilizing identical random seeds and evaluation indicator as the primary experiments, the model’s generalization performance was systematically evaluated across these datasets (results presented in Figure 4). The obtained AUC values were 0.9307, 0.9136, 0.9489, and 0.9064 for miRNA-disease, gene-disease, piRNA-disease, and microbe-disease predictions, respectively. Corresponding AUPR scores reached 0.9125, 0.9089, 0.9521, and 0.9381. These consistently higher performance metrics across diverse biological association tasks demonstrate OFGPMA’s stability and significant generalization capacity. Consequently, these findings provide additional validation for the effectiveness and robustness of the proposed OFGPMA model.

### 3.5 The impact of data imbalance on model performance

**FIGURE 5:**
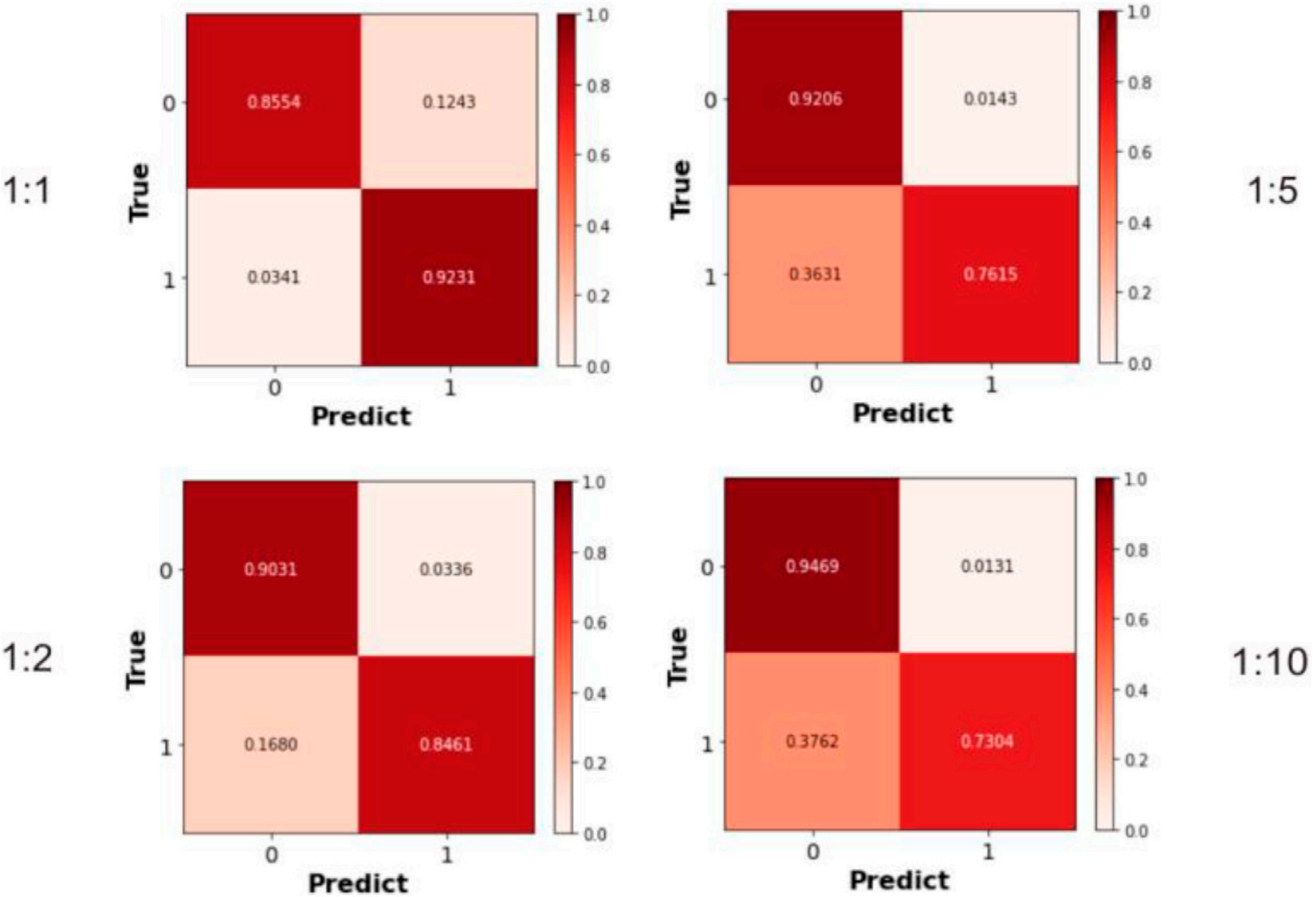
The Confusion matrices calculated under different ratios of positive and negative samples.

Previous experiments employed balanced datasets with equal numbers of positive and negative samples for an initial model evaluation. However, model performance could potentially be influenced by variations in the positive-to-negative sample ratio. To more comprehensively assess OFGPMA’s robustness under class imbalance, we performed five-fold cross-validation on the starBase dataset, specifically testing performance at positive-negative ratios of 1:1, 1:2, 1:5, and 1:10. A visual representation of the confusion matrix is provided in Figure 5, and detailed performance metrics are tabulated in Table 4. Analysis reveals that as the ratio shifts from 1:1 to 1:2, OFGPMA’s average AUC exhibits a gradual increase, potentially attributable to the random seed enhancing model performance. By contrast, the AUPR score showed a significant decline, dropping from 0.9105 to 0.8994. The AUPR metric is frequently utilized to assess classifier performance, particularly under imbalanced data conditions. Although AUPR values experience a significant drop, they remain within a practically acceptable range (Ling et al., 2025; Saito and Rehmsmeier, 2015). As illustrated in Figure 5, a substantial increase in false negatives coincides with a marginal improvement in accuracy, while both recall and precision exhibit considerable declines. Overall, these results suggest that balanced datasets, featuring an equal ratio of positive to negative samples, yield optimal training outcomes, enabling the model to reach peak predictive accuracy.

**TABLE 4:** Performance of OFGPMA under different positive-to-negative ratios on starBase dataset.

Evaluation metrics	Positive: Negative sample ratio			
	1:1	1:2	1:5	1:10
AUC	0.8718	0.8816	0.8753	0.8632
AUPR	0.9105	0.9087	0.9063	0.8994
Precision	0.9211	0.9203	0.9184	0.9103
Recall	0.9015	0.8967	0.8915	0.8834
F1_score	0.9133	0.9211	0.9033	0.8935

### 3.6 Hyperparameter sensitivity analysis

Hyperparameter sensitivity analyses were performed for OFGPMA under controlled conditions, where non-target parameters remained fixed to isolate performance impacts of critical variables.

#### 3.6.1 Effect of the learning rate

**FIGURE 6:**
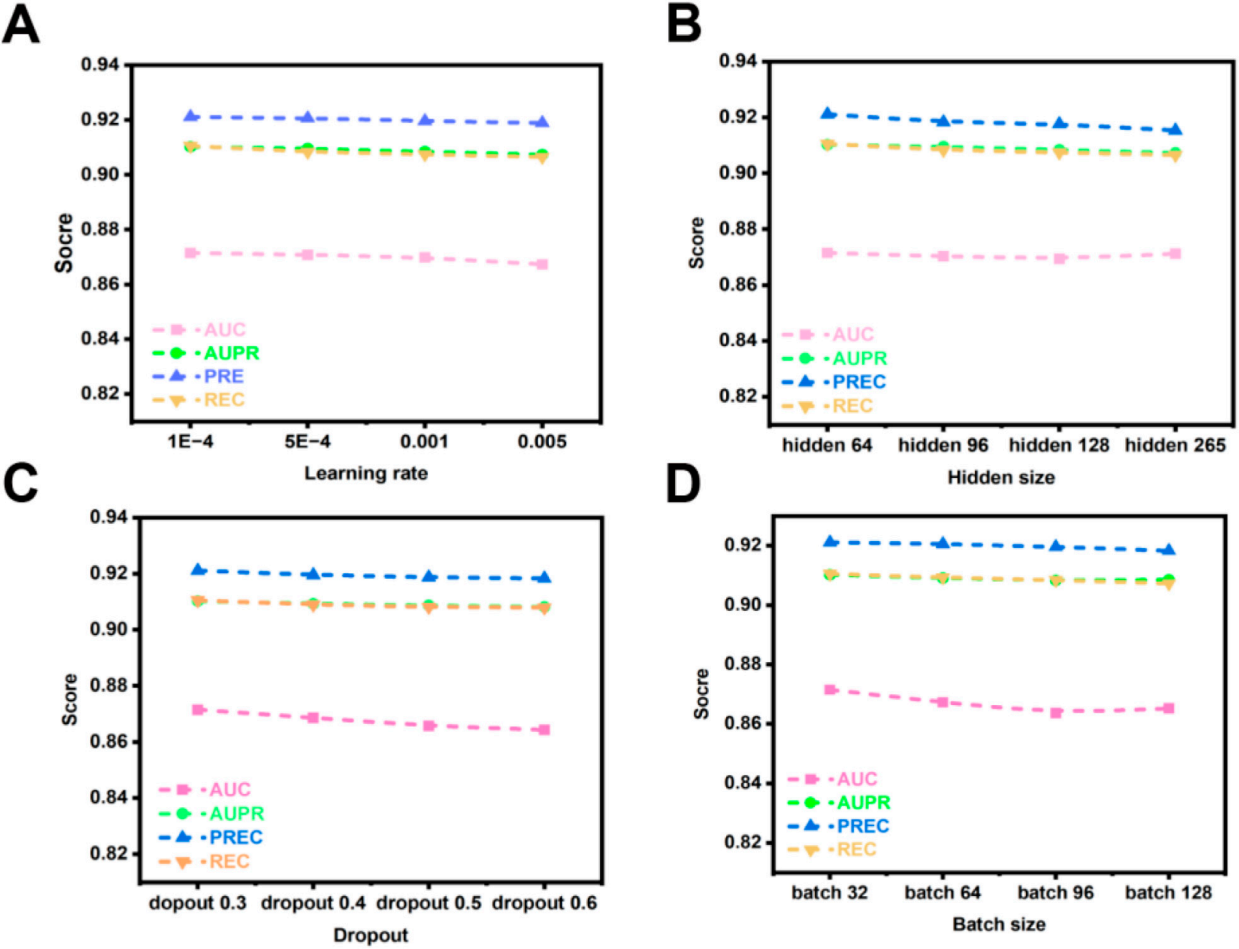
OFGPMA performance of AUC, AUPR, PREC and REC under different hyperparameter. **(A)** denotes learning rate, **(B)** indicates hidden size, **(C)** denotes dropout, **(D)** indicates batch size.

The learning rate, a critical hyperparameter, governs the magnitude of adjustments applied to model weights during optimization. Its value critically influences both the efficiency of the training process and the ultimate performance of the model. Excessively low learning rates impede gradient updates, extending training duration. Conversely, excessively high learning rates risk inducing gradient explosion, which can prevent model convergence. Consequently, investigating the effect of learning rate variation on the OFGPMA model is highly pertinent. Figure 6A demonstrates a progressive decline in OFGPMA’s performance as the learning rate escalates. Experimental findings reveal that a learning rate of 1e-4 yields the optimal model performance, achieving an AUC of 0.8718, AUPR of 0.9105, precision (PREC) of 0.9211, recall (REC) of 0.9015, and F1-score of 0.9133. Therefore, the learning rate for OFGPMA was ultimately fixed at 1e-4.

#### 3.6.2 Effect of the batch size

Batch size represents a crucial hyperparameter in model optimization. While smaller batches can facilitate model convergence, they often constrain training speed and scalability. Conversely, larger batches, despite enabling more efficient utilization of available computational resources and enhancing training throughput, may detrimentally affect model generalization capability [59, 60]. To investigate the influence of batch size on OFGPMA’s performance, we evaluated values within the set {32, 64, 96, 128}. Performance metrics, as depicted in Figure 6D, exhibit a declining trend with increasing batch size. A comprehensive analysis of experimental outcomes and model efficacy led to the selection of a batch size of 32 for conducting subsequent experiments on the starBase dataset.

#### 3.6.3 Effect of the hidden size

Furthermore, the dimensionality of latent representations (hidden size) critically influences model behavior. Insufficient hidden dimensions may result in underfitting, whereas excessive dimensions heighten overfitting risks and prolong training duration. To address this, we systematically evaluated OFGPMA’s performance across hidden sizes spanning {64, 96, 128, 256}. As evidenced in Figure 6B, the model achieves peak performance on the starBase dataset with a hidden dimension of 64.

#### 3.6.4 Effect of the dropout

As a regularization technique, dropout mitigates overfitting by stochastically deactivating neural units during training. For OFGPMA, dropout rates were evaluated across {0.3, 0.4, 0.5, 0.6, 0.7}, with performance outcomes detailed in Figure 6C. Optimal model efficacy was observed at a dropout probability of 0.3.

### 3.7 Ablation experiment

The embedding representations for pseudogenes and miRNAs in OFGPMA are learned through two core components: the Optimal Frequency Discovery (OFD) module and the Graph Representation Learning (GRL) module. To assess the contributions of these modules, ablation studies were executed on the starBase dataset. Three model variants are subsequently defined for comparative analysis:

• OFGPMA w/o OFD: a variant without the optimal frequency discovery (OFD) module.
• OFGPMA-RWR: a variant that incorporating random walk with restart (RWR) for subgraph sampling *in lieu* of the enclosing subgraph extraction strategy.
• OFGPMA-ES: a variant that implementing enclosing subgraph extraction as a substitute for random walk with restart (RWR)-based subgraph sampling.

**FIGURE 7:**
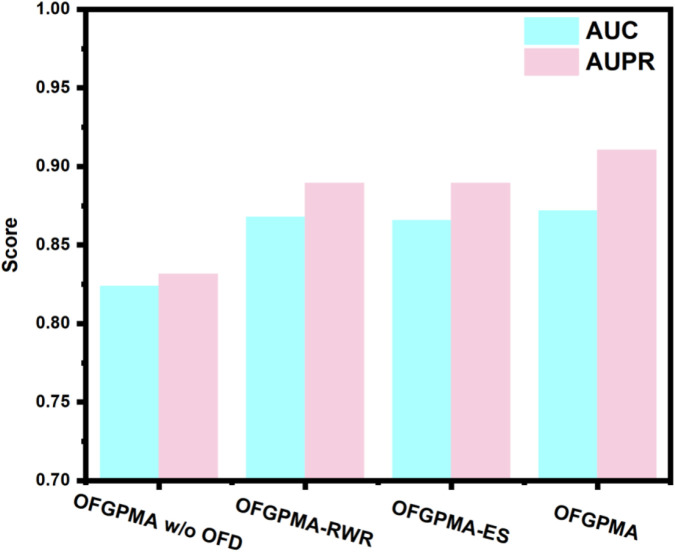
Ablation experiment performance for OFGPMA and three model variants.

As shown in Figure 7, results suggest that the optimal frequency discovery (OFD) module and the graph guidance representation learning (GRL) module are integral components for OFGPMA. Specifically, OFGPMA demonstrates superior performance on every metric. OFGPMA-RWR ranks second overall, while OFGPMA without OFD performs the worst of all models. This might be because the optimal frequency discovery module successfully captured the high-frequency and low-frequency energy information of the graph, thereby significantly enhancing performance and further verifying the effectiveness of OFD. Removing OFD (w/o OFD) led to the largest performance drop, underscoring the importance of frequency analysis. Using only RWR or enclosing subgraphs (OFGPMA-RWR/ES) resulted in intermediate performance, highlighting the value of combining global and local perspectives.

OFGPMA outperforms OFGPMA-RWR and OFGPMA-ES, mainly due to its adoption of a more efficient full-graph information capture strategy, which enriches the structural semantic information. Ablation studies reveal that the newly introduced OFD plays a critical role in OFGPMA’s effectiveness. The incorporation of Random Walk with Restart (RWR) and enclosing subgraph extraction also helped boost prediction performance.

### 3.8 Case study

To evaluate the performance of the OFGPMA method in predicting pseudogene-miRNA interactions, we randomly selected two widely studied pseudogenes, RPLP0P2 and MTND4P12, from the ground truth of the starBase database. For every pseudogene analyzed, we deliberately masked its known miRNA interactions during testing. The remaining candidate miRNAs were then sorted in descending sequence using OFGPMA’s computed prediction scores. Finally, we selected the top-ranked miRNAs and verified their prediction accuracy through the starBase database.

Regarding the pseudogene MTND4P12 (Table 5), two prediction errors occurred. This oncogenic pseudogene exhibits dysregulation in cutaneous melanoma, functioning as a competing endogenous RNA (ceRNA) to upregulate the oncogene AURKB [44]. Notably, Hsa-let-7e-5p is a likely regulatory target of MTND4P12, with both entities showing correlated expression patterns in this malignancy.

**TABLE 5:** Evidence identifies the top 10 miRNAs linked to pseudogenes RPLP0P2 and MTND4P12.

Rank	MTND4P12		RPLP0P2	
1	hsa-let-7e-5p	Confirmed	hsa-miR-34c-5p	Confirmed
2	hsa-let-7d-5p	Confirmed	hsa-miR-195-5p	Confirmed
3	hsa-let-7f-5p	Confirmed	hsa-miR-320d	Confirmed
4	hsa-let-7c-5p	Confirmed	hsa-let-7b-5p	Confirmed
5	hsa-miR-448	Unconfirmed	hsa-miR-15a-5p	Unconfirmed
6	hsa-let-7d-5p	Confirmed	hsa-miR-503-5p	Confirmed
7	hsa-miR-17-5p	Unconfirmed	hsa-miR-3619-5p	Confirmed
8	hsa-let-7b-5p	Confirmed	hsa-miR-16-5p	Confirmed
9	hsa-let-7g-5p	Confirmed	hsa-miR-146a-5p	Unconfirmed
10	hsa-let-7a-5p	Confirmed	hsa-miR-195-5p	Confirmed

Regarding pseudogene RPLP0P2 (Table 5), our model generated three erroneous predictions. This non-coding sequence is implicated in oncogenesis, particularly lung adenocarcinoma and colorectal carcinoma. Prior research indicates that suppressing RPLP0P2 expression reduces malignant cell proliferation and impairs cellular adhesion mechanisms (Chen et al., 2018; Yuan et al., 2021).

## 4 Conclusion

This study proposes an Optimal Frequency Graph Representation Learning Approach (OFGPMA) for predicting pseudogenes-miRNAs association. The model consists of two core modules: the optimal frequency discovery module and the graph representation learning module. In the optimal frequency discovery module, the high-frequency and low-frequency energy information of the given pseudogene-miRNA bipartite graph is extracted through Rayleigh quotient pooling and Chebyshev pooling. These high- and low-frequency spectral components are subsequently integrated into a unified graph representation, amplifying the representational capacity of the graph neural network (GNN). Next, in the graph representation learning module, we extract local closed subgraphs for pseudogenes and global random walk restart (RWR) information for miRNAs based on the fused graph. Subsequently, the extracted closed subgraphs and global graphs are input into a two-layer graph convolutional network (GCN) to obtain node representations. Additionally, to align the high-frequency and low-frequency energy information, a loss function between the high-frequency and low-frequency energy information is introduced to meet the requirements of specific biological hypotheses. Pseudogene-miRNA interaction probabilities are derived from the synthesized representations via MLP transformation. Validation on the starBase dataset confirms OFGPMA’s significant performance advantage. Furthermore, case investigations reveal OFGPMA’s predictive power extends to undocumented pseudogene-miRNA relationships, multiple of which show starBase-documented biological validation.The advantages of OFGPMA are mainly reflected in the following three aspects: First, by learning graph information at different frequencies, it greatly enhances the representation learning ability of GNN; second, by combining local closed subgraphs and global RWR to extract topological structure information of the graph, it requires neither domain expertise nor external datasets, significantly boosting the model’s scalability; third, experimental results show that OFGPMA exhibits superior transfer generalization ability in predicting the associations between miRNAs and other biological entities, providing great potential for its application in other related fields. Despite these achievements, there are still some issues that need to be addressed. Existing datasets documenting pseudogene-miRNA interactions remain sparse, constraining model interpretability and predictive performance. Additionally, the current model only considers the structural information in the pseudogene-miRNA network and ignores the roles of other biomolecules closely related to pseudogenes and miRNAs (such as genes and transcription factors). In future work, incorporating these biomarkers could enable development of more comprehensive biological knowledge graphs, capturing deeper semantic relationships to enhance prediction accuracy of pseudogene-miRNA interactions.

## Data Availability

The original contributions presented in the study are included in the article/Supplementary Material, further inquiries can be directed to the corresponding author.
