# Supplementary material for: OFGPMA: Optimal frequency graph representation learning for pseudogene and miRNA association prediction
Source: Front Genet. 2025 Nov 26;16:1643921. doi: 10.3389/fgene.2025.1643921 (PMC12688278; doi:10.3389/fgene.2025.1643921)
Supplement: Supplementary file 1 [file Table2.docx]

*Supplementary Material*

## **1 Node representation**

MiRNA sequence data is represented as a string composed of four nucleotides. In this paper, we use k-mer to represent miRNA sequences as a 64-dimensional feature vector, where $k = 3$. Similarly, pseudogenes are processed in the same way. The final feature matrix dimension of pseudogenes (P) is 444$\times$64, and that of miRAN (M) is $173\times64$. Then, PMA matrix is constructed and its dimension is $444\times173$. In PMA matrix, if pseudogene *i* associate with miRNA *j*, $PMA(i, j) = 1$, else $PMA(i, j) = 0$. Finally, the PMA network A and node feature X are calculated as:

$X=\left( \frac{P}{M} \right)$ (1)

$A=(\frac{0}{PMA^{T}}\frac{PMA}{0})$ (2)

### **2 Rayleigh pooling**

$E\left( t \right)=\sum_{j=1}^{k} \frac{X_{j}}{\sum_{i=1}^{n} X_{i}}$ (3)

${RQ}_{high}=\mu\int_{0}^{\vartheta} E\left( t \right)dt+(1-\mu)\int_{\vartheta}^{\lambda_{n}} E\left( t \right)dt$ (4)

$H_{RQ}=MLP({RQ}_{high})$ (5)

where $\lambda_{n}=(\lambda_{1}, \lambda_{2}, \ldots, \lambda_{n})$ is the ascendingly ordered eigenvalues (i.e., spectral frequencies) of the Laplacian matrix $L$. $\vartheta$ is a learnable filtering parameter used to distinguish and filter high-frequency energy information from low-frequency energy information. $\int_{0}^{\vartheta} E\left( t \right)dt$ represents the summation of low-frequency energy information, and $\int_{\vartheta}^{\lambda_{n}} E\left( t \right)dt$ represents the summation of high-frequency energy information. $\mu$ is a learnable parameter.

### **3 Chebyshev Pooling**

$f\left( L \right)=\frac{1}{2}c_{0}I+\sum_{k=1}^{K} c_{0}T_{k}\left( L \right)$ (6)

$T_{k}\left( L \right)=\frac{1}{\tau}\left( L-I \right)T_{k-1}\left( L \right)-T_{k-2}\left( L \right)$ (7)

where $T_{k}\left( L \right)$ is the Chebyshev polynomial after translation scaling, $T_{0}\left( L \right)=I$, $T_{1}\left( L \right)=L$ and $c_{0}$ denotes Filter coefficients, $\tau$ is learned parameter. *I* is an identity matrix. Low-order polynomials K: mainly capture low-frequency signals, corresponding to the eigenvectors of small eigenvalues. By adjusting the polynomial order K, Chebyshev polynomials can flexibly control the receptive field and retain the smooth low-frequency features. The low-frequency energy signal of the final PMA graph can be expressed as:

$H_{CWT}=f\left( L \right)*X$ (8)

## **4** **Encoder layer**

To cover the neighborhood information of both the local encolsing subgraph and the RWR global graph, we merge the two graphs $G_{m}^{k}$ and $G_{RWR}$:

$V^{new}=V_{m}\cup V_{RWR}$ (9)

$E^{new}=E_{m}\cup E_{RWR}$ (10)

$G^{new}=G_{m}^{k}\cup G_{RWR}$ (11)

where $V_{m}\in G_{m}^{k}$ and $V_{RWR}\in G_{RWR}$ are node set, $E_{m}\in G_{m}^{k}$ and $E_{RWR}\in G_{RWR}$ are edge set.

Next, we use two layers of GCN to learn topological features for $G_{m}^{k}$ and $G_{RWR}$:

$X=Embeddings$ (12)

$Z^{p}=\sigma(\tilde{D}^{-\frac{1}{2}}\tilde{A_{m}}\tilde{D}^{-\frac{1}{2}}\sigma\left( \tilde{D}^{-\frac{1}{2}}\tilde{A_{m}}\tilde{D}^{-\frac{1}{2}}XW_{m}^{0} \right)W_{m}^{1})$ (13)

$Z^{RWR}=\sigma(\tilde{D}^{-\frac{1}{2}}\tilde{A_{RWR}}\tilde{D}^{-\frac{1}{2}}\sigma\left( \tilde{D}^{-\frac{1}{2}}\tilde{A_{RWR}}\tilde{D}^{-\frac{1}{2}}XW_{RWR}^{0} \right)W_{RWR}^{1})$ (14)

$Z^{new}=\varphi Z^{m}+{\omega Z}^{RWR}$ (15)

where $Z^{m}$ and $Z^{RWR}$ denote the embeddings for $G_{m}^{k}$ and $G_{RWR}$. $\tilde{A}=A+I$, $I$ is the identity matrix. $A$ denotes adjacency matrix and $\tilde{D}$ is the degree matrix of $\tilde{A}$. $W^{0}$ and $W^{1}$ are learnable weight parameters. $\sigma(*)$ is the sigmoid activation function. $\varphi$ and $\omega$ are the learnable coefficients.

## **5 Evaluation criteria**

In OFGPMA. we employ frequently five evaluation metrics to evaluate its performance, including AUC, AUPR, PREC, REC and F1-score. AUC denotes the area under the Receiver Operating Characteristic (ROC) curve, AUPR indicates the area under the Precision-Recall (PR) curve, PREC refers to precision, and REC stands for recall., respectively.

$Precision(Prec. )=\frac{TP}{FP+TP}$ (16)

$Recall=\frac{TP}{TP+FN}$ (17)

$F1-score=\frac{2\times Precision\times Recall}{Precision+Recall}$ (18)

where TP, FP, TN, and FN refers to the numbers of true positives, false positives, true negatives, and false negatives samples, respectively.

## **6 Robustness experimental data processing**

For gene and piRNA, we use the k-mer algorithm to extract node features. In microbe-disease association prediction, in disease, following the common practice in the disease field, we extracted the semantic similarity of diseases as the initial features of the nodes, for microbes, we adopt Gaussian interaction profile kernel similarity as the extraction method for the node features of microbes. The specific data processing method follows the relevant procedures proposed by Zheng et al (Zheng et al., 2023).

**1. Detailed Description of Model Adaptation for Different Association Types​​**

The core architecture of OFGPMA remains consistent across different biological association prediction tasks (miRNA-disease, gene-disease, piRNA-disease, microbe-disease). The adaptation primarily occurs at the ​**​input data layer​**​-specifically, in the construction of the bipartite graph and the initial node feature engineering-while the core Optimal Frequency Discovery (OFD) and Graph Representation Learning (GRL) modules are applied generically. The following table summarizes the key adaptations for each dataset:

| **Biological Association Type** | **Node Types (Bipartite Graph)** | **Node Feature Extraction Method (Adaptation Step)** | **Core OFGPMA Modules Applied** |
| --- | --- | --- | --- |
| ​**​miRNA-Disease​**​ | miRNA Nodes, Disease Nodes | ​**​miRNAs​**​: k-mer frequency vectors (k=3). ​**​Diseases​**​: Semantic similarity between diseases, calculated based on the Medical Subject Headings (MeSH) descriptors, was used as feature vectors. | Unchanged (OFD + GRL) |
| ​**​Gene-Disease​**​ | Gene Nodes, Disease Nodes | ​**​Genes​**​: k-mer frequency vectors (k=3) from gene sequences. ​**​Diseases​**​: Semantic similarity feature vectors. | Unchanged (OFD + GRL) |
| ​**​piRNA-Disease​**​ | piRNA Nodes, Disease Nodes | ​**​piRNAs​**​: k-mer frequency vectors (k=3). ​**​Diseases​**​: Semantic similarity feature vectors. | Unchanged (OFD + GRL) |
| ​**​Microbe-Disease​**​ | Microbe Nodes, Disease Nodes | ​**​Microbes​**​: Gaussian Interaction Profile (GIP) kernel similarity, which calculates similarity based on the topological structure of the known association network, was used as node features. ​**​Diseases​**​: Semantic similarity feature vectors. | Unchanged (OFD + GRL) |

The choice of feature extraction method for each node type is a standard practice in the respective computational biology fields. For instance, k-mer frequency is effective for capturing sequence-level characteristics of RNAs and genes. Semantic similarity is well-established for representing diseases in a structured vector space. The GIP kernel similarity is particularly useful for microbes where sequence data might be less informative than interaction patterns. By transforming the raw data of each entity type into a consistent numerical feature vector format, we ensure that the input to the OFGPMA model is a well-defined bipartite graph with meaningful node attributes, regardless of the specific biological entities involved. The core model then processes this graph to learn the high/low-frequency components and global/local topological features relevant to the association network.

Zheng, K., Zhang, X. L., Wang, L., You, Z. H., Ji, B. Y., Liang, X., & Li, Z. W. (2023). SPRDA: a link prediction approach based on the structural perturbation to infer disease-associated Piwi-interacting RNAs. *Briefings in Bioinformatics*, *24*(1). https://doi.org/10.1093/bib/bbac498
